# 3,3′-Diindolylmethane Augments 5-Fluorouracil-InducedGrowth Suppression in Gastric Cancer Cells through Suppression of the Akt/GSK-3*β* and WNT/Beta-Catenin

**DOI:** 10.1155/2023/8268955

**Published:** 2023-02-04

**Authors:** Cong Shan Li, Thi Van Nguyen, Ok Hee Chai, Byung Hyun Park, Ju-Seog Lee, Soo Mi Kim

**Affiliations:** ^1^Department of Physiology, Institute for Medical Sciences, Jeonbuk National University Medical School, Jeonju 54907, Republic of Korea; ^2^Department of Anatomy, Institute for Medical Sciences, Jeonbuk National University Medical School, Jeonju 54907, Republic of Korea; ^3^Department of Biochemistry, Jeonbuk National University Medical School, Jeonju, Republic of Korea; ^4^Department of Systems Biology, The University of Texas MD Anderson Cancer Center, Houston, TX 77030, USA

## Abstract

Gastric cancer (GC) is one of the most lethal cancers in South Korea, and it is a cancer of concern worldwide. 5-fluorouracil (5-Fu) is commonly used as the first-line therapy for advanced GC; however, its side effects often limit the dosage range and impair patients' quality of life. Due to the limitations of current chemotherapy, new anticancer therapies are urgently needed. 3,3′-diindolylmethane (DIM) has been reported to have the ability to protect against various types of cancer. Our study aimed to elucidate the anticancer effect of DIM in GC when treated with the chemotherapeutic agent 5-Fu. In our results, combined treatment with DIM and 5-Fu resulted in higher apoptosis and lower cell proliferation than treatment with 5-Fu in SNU484 and SNU638 cell lines. Furthermore, when DIM and 5-Fu were administered together, cell invasion was diminished by mediated E-cadherin, MMP-9, and uPA; p-Akt and p-GSK-3*β* levels were reduced more significantly than when 5-Fu was administered alone. Moreover, in the Wnt signaling pathway, combined treatment of DIM and 5-Fu diminished *β*-catenin levels in the nucleus and inhibited cyclin D1and c-Myc protein levels. The Akt inhibitor, wortmannin, further inhibited the levels of *β*-catenin and c-Myc that were inhibited by DIM and 5-Fu. Furthermore, an animal xenograft model demonstrated that DIM combined with 5-Fu considerably reduced tumor growth without any toxic effects by regulating the Akt/GSK-3*β* and *β*-catenin levels. Our findings suggest that DIM significantly potentiates the anticancer effects of 5-Fu by targeting the Akt/GSK-3*β* and WNT/*β*-catenin because the combination therapy is more effective than 5-Fu alone, thereby offering an innovative potential therapy for patients with GC.

## 1. Introduction

Gastric cancer (GC) has a high incidence rate and is one of the most lethal cancers worldwide [1]. Moreover, GC is a prominent mortality malignant neoplasm in South Korea [2]. Although the morbidity of GC has recently declined globally, it remains a major killer worldwide [3]. Due to the lack of an early GC diagnostic index, only 28% of patients with GC can be diagnosed in the local stage, and more than 62% of patients were initially discovered to have regional or distant GC [4]. Thus, the 5-year survival rate of patients with GC is merely 37.9% [5]. Surgery remains the preferred method for treating patients with GC because of the limitations of chemotherapy [6]. However, many patients are diagnosed late with advanced-stage disease because of the low rate of early diagnosis [7]. Consequently, most patients with GC miss the surgical time window; the treatment for late-stage is the combination of neoadjuvant chemotherapy, radiotherapy, specific-targeted therapy, and others [7]. Therefore, GC remains a serious burden on global health because therapies for this disease are limited, and novel therapies are desperately needed.

5-fluorouracil (5-Fu) acts by interrupting the action of DNA replication [8]. Over 50 years, 5-Fu has been extensively used as systemic combination chemotherapy in treating colorectal [9], gastrointestinal [10], anal [11], breast [12], head, and neck cancers [13, 14]. Although 5-Fu is recommended as a principal chemotherapeutic reagent for GC [15], various acute problems, such as dose-limiting toxicity, low efficient conversion rate, drug resistance, and serious side effects, will decrease its efficacy [16, 17]. Thus, developing natural substances that can alleviate the side effects of the existing anticancer action and increase the efficiency of 5-Fu are imperative.

3,3′-diindolylmethane (DIM) is found as phytochemicals in fruits and vegetables [18] and has excellent proven anticancer capabilities [19–24]. DIM prevents cancer growth and metastasis as well as induces autophagy and cell apoptosis in colon [25], pancreatic cancer [26], breast cancer [27], endometrial cancer [28], and GC [29, 30]. Furthermore, DIM plays an essential role in regulating many pathways, including the Akt [31], Wnt [24, 25], and Hippo [30] signaling pathways. In our previous study, we found that DIM potentiated paclitaxel-induced anticancer effects by inactivating Akt and FOXM1 in GC cells [29]. Hence, DIM could be used as an enhancive drug to reduce the limitations of existing anticancer drugs; the addition of DIM may promote the antitumor efficacy of chemotherapeutic agents in GC.

Akt is a serine/threonine kinase and acts as a key player in the phosphoinositide 3-kinase (PI3K)/Akt signaling pathway involved in normal cellular processes [32]. Akt is often highly activated in many cancers [33] and functions as a central point in various signaling pathways [34]. PI3K/Akt pathway has been suggested to be associated with cell invasion, autophagy, and apoptosis regulation in GC [35]. Moreover, Yu et al. have demonstrated that Akt is involved in the chemoresistance that causes drug resistance in GC [36, 37]. Hence, targeting Akt seems to be an important approach for preventing and treating GC. The Wnt pathway elicits a pivotal role in GC [38]. Approximately 30% of gastric adenocarcinomas show a direct correlation with *β*-catenin nuclear localization through the Wnt signaling pathway [39]. Although DIM can regulate cell proliferation via Akt [29] or *β*-catenin [40], whether DIM can enhance the effects of chemotherapeutic drugs on GC via the Akt and Wnt signaling pathways remains uncertain. Therefore, our study was designed to investigate the effects of DIM combined with 5-Fu and the probable underlying biological mechanisms in GC. We hypothesize that combination treatment of DIM and 5-Fu has a strong and safe inhibitory effect on GC and effectively improves the susceptibility of GC to DIM by inhibiting Akt/GSK-3*β* and *β*-catenin. Therefore, DIM may be a feasible targeted therapy for GC.

## 2. Materials and Methods

### 2.1. Cell Culture and Experimental Reagents

SNU484 and SNU638 were acquired from the Korean Cell Line Bank (Seoul National University, Korea). RPMI-1640 medium (Gibco, Grand Island, NY, USA) containing 10% fetal bovine serum (FBS) (Welgene Gold Serum, Gyeongsan-si, Republic of Korea) and 1% penicillin (Sigma-Aldrich, St. Louis, USA) were used for cell culture. Antibodies, such as glyceraldehyde 3-phosphate dehydrogenase (GAPDH), cleaved-PARP, cleaved-caspase-9, PARP, caspase 9, cyclin D1, Akt, p-Akt, GSK-3*β*, *β*-catenin, p-*β*-catenin, cyclin D1, and lamin B, and secondary antibodies against rabbit and mice were acquired from Cell Signaling Technology, Inc. (Danvers, Massachusetts, USA); E-cadherin, uPA, MMP-9, and c-Myc were bought from Santa Cruz Biotechnology, Inc. (Santa CRUZ, CA, USA). Wortmannin and 5-FU were acquired from Sigma-Aldrich (St. Louis, MO, USA) and DIM was acquired from LKT Laboratories (St. Paul, MN, USA).

### 2.2. Cell Viability Assay

The cells were seeded in 96-well plates. Each well contained approximately 10^4^ cells. After 48 h of DIM (30 *μ*M) and 5-Fu (10 *μ*M) treatment, each well containing 10 *μ*L of EZ-Cytox (DoGenBio, Seoul, South Korea) was diluted with 100 *μ*L of RPMI-1640. Then, a microplate reader was used to determine the data (450 nM, Bio-Tek, Winooski, VT, USA).

### 2.3. Soft Agar Colony Formation Assay

Moreover, 1% agarose gel was dissolved and added to a 6-well dish until solidification. Following this, 0.7% agarose gel was gently mixed with 1 × 10^5^ cells in 2 × RPMI-1640 media and 20% FBS plus 2% antibiotics and covered with medium with or without drug, respectively. The dishes were incubated for 3 weeks and fed fresh medium with or without drug twice or three times a week. Colonies with more than 30 cells were counted as one positive colony, and images were taken using a microscope.

### 2.4. Annexin V–FITC Analysis

An Annexin V–FITC Assay Kit (Becton Dickinson Biosciences, CA, USA) was used according to the manufacturer's recommended procedure. After 48 h of DIM and 5-Fu treatment, the cells were washed twice with ice-cold DPBS and further resuspended in the annexin-binding buffer containing annexin V-FITC and propidium iodide (PI) solution. Following the incubation of stained cells in a CO_2_ incubator at 37°C for 30 min, flow cytometry analysis was performed immediately (Becton Dickinson, New York, NY).

### 2.5. Cell Cycle Analysis

To analyze the effect of drugs on the cell cycle, SNU484 and SNU638 were cultured in 60 mm dishes and incubated with DIM and 5-Fu for 48 h. After collecting the cells, they were rinsed twice with ice-cold DPBS and soaked for 2 h in 75% ethanol. Subsequently, the cells were washed with DPBS to remove the ethanol. The cell cycle counts were estimated by incubating at 37°C in a CO_2_ incubator for 15 min with RNase, which was followed by nucleus staining with propidium iodide (Sigma Chemical, St. Louis, MO, USA). Then, cell counts were further analyzed using CytExpert analysis software (Beckman Coulter, Indianapolis, Indiana, USA).

### 2.6. Migration Assay

After attaching the cells (6-well plate), a straight line was scraped on the monolayer cells, and the floating debris was removed using DPBS. Cells were further applied with DIM and/or 5-Fu for 48 h with 5% FBS. Moreover, images were taken from a microscope (IX71; Olympus, PA, USA), and the vertical distance of each scratch between two sides was measured after at least five cells were randomly selected. The closure area was measured after 24 h and 48 h.

### 2.7. Western Blotting

Proteins were extracted from mouse tumor tissue or cell lysate after 48 h of treatment with DIM, 5-Fu, or a combination treatment. Wortmannin (5 *μ*M) was pretreated for 2 h, and samples were collected 48 h after DIM, 5-Fu, or a combination treatment. Whole proteins were lysed and separated cytoplasmic and nuclear proteins using NE-PER™ Nuclear and Cytoplasmic Extraction Reagents (Thermo, Meridian Rd, Rockford, USA). Extracts were placed into lyse and then centrifuged (13,200 rpm for 30 min). Protein levels were measured by BSA protein assay kit (Pierce Biotechnology, Inc., Rockford, IL, USA). Proteins (30 *μ*g) were resolved using SDS-PAGE gels (8% or 12%) and transferred onto polyvinylidene difluoride membranes (Bio-Rad, CA, USA). Specific primary antibodies were incubated on the membranes overnight at 4°C; afterward, HRP-conjugated secondary antibodies were incubated for at least 2 h at 4°C. The Immobilon Western Chemiluminescent HRP Substrate (Millipore Corporation, Burlington, MA, USA) was used to capture chemiluminescent images by the Amersham Imager 600 (GE Healthcare Bio-Sciences AB, Uppsala, Sweden). The primary antibodies included Bax, Bcl2, cleaved-caspase3, cleaved-PARP, cleaved-caspase-9, cleaved-caspase-7, caspase-3, PARP, caspase-9, caspase-7, uPA, MMP-9, E-cadherin, GSK-3*β*, p-GSK-3*β*, p-Akt, Akt, *β*-catenin, p-*β*-catenin, cyclin D1, c-Myc, lamin B, and GAPDH.

### 2.8. RNA Isolation and RT-PCR

Total RNA was prepared as previously described [41]. The E-cadherin primer sequences used were 5′-GGATTGCAAATTCCTGCCATTC-3′ and its antisense (5′-AACGTTGTCCCGGGTGTCA-3′); MMP-9 sense (5′-GACCTCAAGTGGCACCACCA-3′) and antisense (5′-GTGGTACTGCACCAGGGCAA-3′); GAPDH sense (5′-GTCTCCTCTGACTTCAACAGCG-3′) and antisense (5′-ACCACCCTGTTGCTGTAGCCAA-3′).

### 2.9. In Vivo Studies

*In vivo* studies were performed according to the guidelines by the Institutional Animal Care and Use Committee (IACUC) of the University of Pennsylvania (USA) and those authorized by the IACUC of Jeonbuk National University (#CBNU2017-0001, 3 January 2017). Immunodeficient mice (4 weeks, male, SPF/VAF) were purchased (Orient Bio, Deajeon, South Korea) and acclimatized to conditions for 1 week before the tumor cells inoculation. Each mouse was subcutaneously injected with Matrigel (3.5 × 10^6^ human GC cell SNU484/0.1 mL) into the one-side flank. The mice were separated into three groups (*n* = 5 in each) as follows: (i) control group (intraperitoneally injected with 50 *μ*L of DPBS every 2 days); (ii) 5-Fu group (intraperitoneally injected with 25 mg/kg of 5-Fu in 50 *μ*L of DPBS every 2 days); (iii) DIM and 5-Fu combination group (intraperitoneally injected with 25 mg/kg of 5-Fu + 10 mg/kg of DIM in 50 *μ*L of DPBS every 2 days). The formula to calculate the tumor volume was as follows: (width) 2 × length/2. When the tumors grew to approximately 4,000 mm^3^, the experiment was terminated, mice were sacrificed, and samples were collected.

### 2.10. Histology and Immunohistochemistry

For histology, tumor tissues were extracted from mice. Then, the tissues were embedded in paraffin after being fixed in 10% formaldehyde solution, sectioned (4 *μ*m), and further stained with H&E. For immunohistochemistry, deparaffinization and rehydration of the formalin-fixedparaffin-embedded tissue were performed. Next, the slices were incubated overnight at 4°C with the anti-Ki-67 antibody (Invitrogen, Waltham, MA, USA). Antirabbit HRP/DAB IHC kit (Abcam, Cambridge, UK) was used to stop high background, and all images were captured using a light microscope.

### 2.11. Blood Biochemical Assays

After euthanasia, the blood was placed in an EP tube and the serum was separated into another EP tube. Aspartate aminotransferase and alanine aminotransferase levels (AM102-K and AM103-K, Asan Pharmaceutical, Seoul, Korea) were measured to evaluate the liver function. The serum creatinine (Cr) and blood urea nitrogen (BUN) levels were determined to assess kidney function (CR, creatinine serum detection Kit and BUN, ARBOR ASAYS, Michigan, USA).

### 2.12. Statistical Analysis

GraphPad Prism version 7.00 (GraphPad Software, Inc., La Jolla, CA, USA) was used to calculate half-maximal inhibitory concentration (IC50), the drug concentration at which 50% growth inhibition was achieved. One-way analysis of variance with Tukey's posthoc analysis was used as the statistical comparison. The data are expressed as the means ± standard error (SE). *p*-values of <0.05 or <0.01 were used to indicate statistical significance.

## 3. Results

### 3.1. DIM Enhances 5-Fu-Inhibited Proliferation of GC Cells

**Figure 1:**
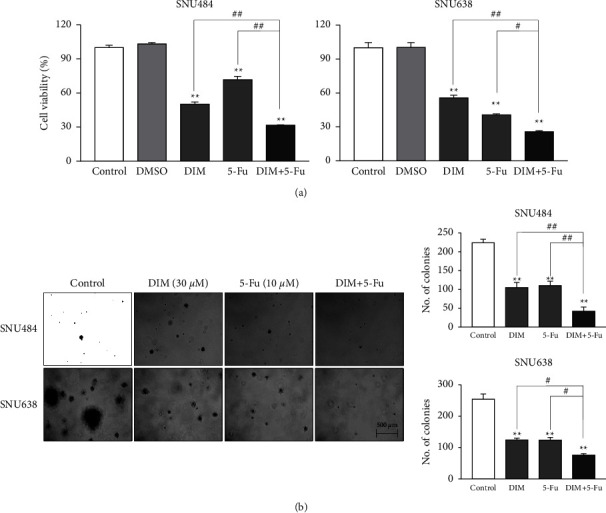
Combination treatment of DIM and 5-Fu diminished the gastric cancer (GC) cell growth. (a) DIM and 5-Fu inhibited cell viability in GC. (b) DIM and 5-Fu combination treatment suppressed cell growth in GC. Cell growth was detected using soft agar colony formation assay. GC cells were grown in agarose gel to form a colony. DIM, 5-Fu, or combination treatment were fed to the colony for 3 weeks. Scale bar = 500 *μ*m. CONT, control; D or DIM, 3,3′-diindolylmethane; 5-Fu, 5-fluorouracil. Each point represents the mean ± standard error (SE). ^*∗*^*p* < 0.05, ^*∗∗*^*p* < 0.01 compared to control; ^#^*p* < 0.05, ^##^*p* < 0.01 compared to DIM + 5-Fu.

We performed WST-1 and colony formation assays using SNU-484 and SNU-638 GC cell lines to measure cell viability. To evaluate the combined effects of DIM and 5-Fu on cell proliferation, SNU484 and SNU638 cells were treated with DIM (0, 10, 20, 30, 40, 50 *μ*M) and 5-Fu (0, 5, 10, 15, 20 *μ*M) and calculated the IC_50_ values of each drug. The IC_50_ values of DIM were 33.84 ± 1.41 and 37.40 ± 0.84 in SNU484 and SNU638 cell lines, while those of 5-Fu were 15.31 ± 0.67 and 6.51 ± 0.48, respectively. Based on these IC_50_ values of each drug, the cells were treated with DIM (30 *μ*M), 5-Fu (10 *μ*M), or a combination treatment (DIM + 5-Fu) for 48 h. Combination treatment of DIM and 5-Fu decreased cell viability by 70% compared with the control group (Figure 1(a)), and the percentage of cell viability inhibition of combination treatment of DIM and 5-Fu was significantly greater than that of the single treatment alone (either DIM alone or 5-Fu alone) in SNU484 cells (Cont: 100% ± 2.18%; DMSO: 103.23% ± 1.23%; DIM: 50.15% ± 1.86%; 5-Fu: 71.74% ± 2.86%; DIM + 5-Fu: 31.70% ± 0.32%) (DIM vs. DIM + 5-Fu: *p* < 0.01; 5-Fu vs. DIM + 5-Fu: *p* < 0.01) and SNU638 cells (Cont: 100% ± 4.53%; DMSO: 100.29% ± 4.33%; DIM: 55.65% ± 2.51%; 5-Fu: 40.61% ± 1.08%; DIM + 5-Fu: 25.86% ± 0.82%) (DIM vs. DIM + 5-Fu: *p* < 0.01; 5-Fu vs. DIM + 5-Fu: *p* < 0.05). Combination treatment of DIM and 5-Fu significantly suppressed colony formation and decreased colony numbers compared with DIM alone or 5-Fu alone in SNU484 cells (Cont: 223.80 ± 9.50; DIM: 105 ± 13.89; 5-Fu: 110 ± 11.67; DIM + 5-Fu: 42 ± 11.09) (DIM vs. DIM + 5-Fu: *p* < 0.01; 5-Fu vs. DIM + 5-Fu: *p* < 0.01) and SNU638 cells (Cont: 254 ± 16.55; DIM: 124 ± 6.04; 5-Fu: 123.75 ± 8.51; DIM + 5-Fu: 75.75 ± 5.36) (DIM vs. DIM + 5-Fu: *p* < 0.05; 5-Fu vs. DIM + 5-Fu: *p* < 0.05) (Figure 1(b)). These results indicated that combined treatment with DIM and 5-Fu inhibited GC cell proliferation more significantly than DIM or 5-Fu alone.

### 3.2. DIM Enhances 5-Fu-Induced Cell Apoptosis and Cell Cycle Arrest in GC Cells

**Figure 2:**
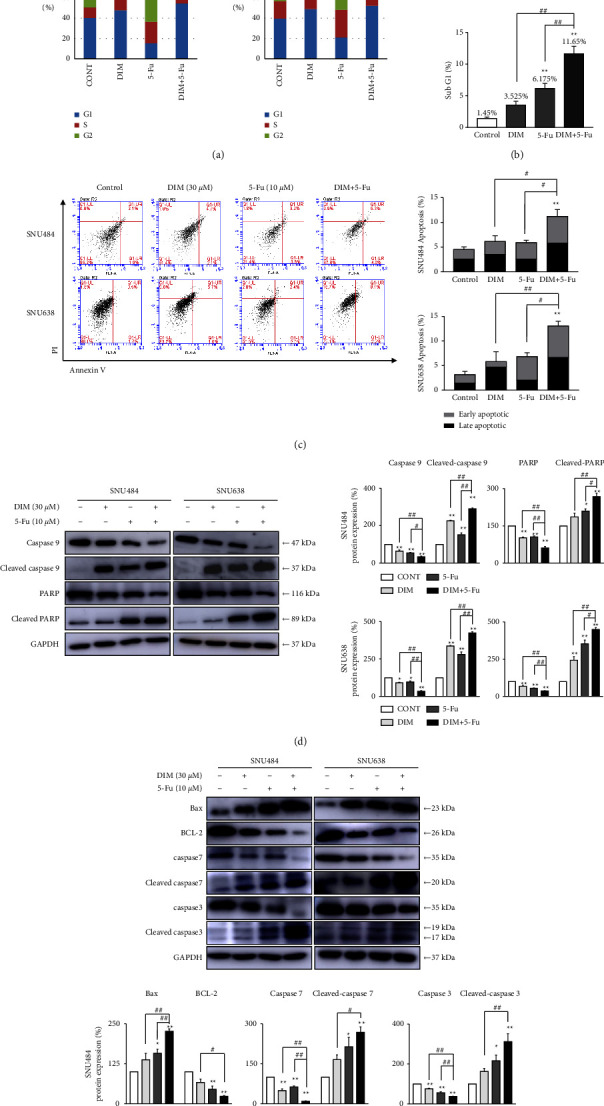
DIM and 5-Fu combination therapy disrupted gastric cancer cell cycle and induced apoptosis. (a) DIM and 5-Fu induced the expression of G1 phase arrest in SNU484 and 638 cell lines. G1 = growth-1 phase; *S* = synthesis phase; G2/M = growth-2 and mitotic phases. (b) The sub-G1 phase was determined by flow cytometry, and then the cell cycle phase distribution was computed. The sub-G1 phase was measured by flow cytometry, and then the distribution of the cell cycle in the sub-G1 phase was estimated. (c) DIM and 5-Fu combination treatment increased the apoptosis/necrotic cells. Histogram of a relative population of apoptotic and necrotic cells was developed and the living cell percentage was quantified to represent the apoptosis and necrosis rate. (d) and (e) DIM and 5-Fu treatment suppressed the apoptotic proteins. GAPDH was implemented throughout the study as an internal control. CONT, control; D or DIM, 3,3′-diindolylmethane; 5-Fu, 5-fluorouracil. Every point corresponds to the mean ± standard error (SE). The gray value of bands was detected using ImageJ. ^*∗*^*p* < 0.05, ^*∗∗*^*p* < 0.01, compared to control; ^#^*p* < 0.05, ^##^*p* < 0.01 compared to DIM + 5-Fu.

Flow cytometry was used to explore the potential impact of DIM and 5-Fuon cell cycle alterations. Additionally, SNU484 and SNU638 populations of gastric cancer cells in the G1 phase were significantly increased by combining DIM and 5-Fu therapy. Researchers posited that a combination treatment of the two drugs induced G1 phase arrest in GC cells (Figure 2(a)). Subsequently, we tested the effects of DIM and 5-Fu on cell apoptosis in GC cells. Since the sub-G1 phase acts as an apoptotic cell marker, we measured the distribution of sub-G1 phase. The percentage of sub-G1 phase increased in the presence of DIM or 5-Fu in SNU484 cells (Cont: 2.5% ± 0.2%; DIM: 4.38% ± 0.92%; 5-Fu: 8.93% ± 1.88%; DIM + 5-Fu: 13.65% ± 3.68%) (DIM vs. DIM + 5-Fu: *p* < 0.05; 5-Fu vs. DIM + 5-Fu: *p*=0.43) and SNU638 cells (Cont: 1.45% ± 0.18%; DIM: 3.53% ± 0.6%; 5-Fu: 6.18% ± 0.79%; DIM + 5-Fu: 11.65% ± 1.18%) (DIM vs. DIM + 5-Fu: *p* < 0.01; 5-Fu vs. DIM + 5-Fu: *p* < 0.01) (Figure 2(b)). Combination treatment of DIM and 5-Fu significantly enhanced sub-G1 phase by 4.72%–9.27% compared with DIM or 5-Fu alone. Simultaneously, we performed annexin V–fluorescein isothiocyanate (FITC) to directly explore the alteration of apoptotic effect in GC cells. The number of cells in the Q1-LR region determines early apoptotic cells, whereas the Q1-UR region contains late apoptotic and necrotic cells. DIM and 5-Fu noticeably improved the ratio of apoptotic effect compared with single treatment of DIM or 5-Fu alone in SNU484 cells (Cont: 1.97% ± 0.03%; DIM: 2.63% ± 0.80%; 5-Fu: 3.33% ± 0.42%; DIM + 5-Fu: 5.43% ± 0.74% of early-stage apoptotic cells; DIM vs. DIM + 5-Fu: *p* < 0.05, 5-Fu vs. DIM + 5-Fu: *p*=0.13) (Cont: 2.57% ± 0.47%; DIM: 3.5% ± 0.69%; 5-Fu: 2.57% ± 0.41%; DIM + 5-Fu: 5.77% ± 0.84% of late-stage apoptotic/necrotic cells; DIM vs. DIM + 5-Fu: *p*=0.12, 5-Fu vs. DIM + 5-Fu: *p* < 0.05) and SNU638 cells (Cont: 1.73% ± 0.14%; DIM: 1.1% ± 0.23%; 5-Fu: 4.83% ± 1.29%; DIM + 5-Fu: 6.45% ± 2.59% of early-stage apoptotic cells; DIM vs. DIM + 5-Fu: *p* < 0.05, 5-Fu vs. DIM + 5-Fu: *p*=0.84) (Cont: 1.43% ± 0.62%; DIM: 4.7% ± 1.9%; 5-Fu: 2.02% ± 0.93%; DIM + 5-Fu: 6.63% ± 2.05% of late-stage apoptotic/necrotic cells; DIM vs. DIM + 5-Fu: *p*=0.78, 5-Fu vs. DIM + 5-Fu: *p* < 0.05) (Figure 2(c)). Furthermore, we measured apoptosis-related proteins, using western blotting. Combination treatment of DIM and 5-Fu downregulated Bcl-2, caspase-3, caspase-7, PARP, and caspase-9 and on the other hand, upregulated the cleaved form of Bax, caspase-3, caspase-7, PARP, and caspase-9 compared with DIM or 5-Fu alone (Figures 2(d) and 2(e)). Our findings indicated that combined treatment of DIM and 5-Fu greatly enhances apoptosis in GC cells.

### 3.3. DIM Enhances 5-Fu-Inhibited Migration of GC Cells

**Figure 3:**
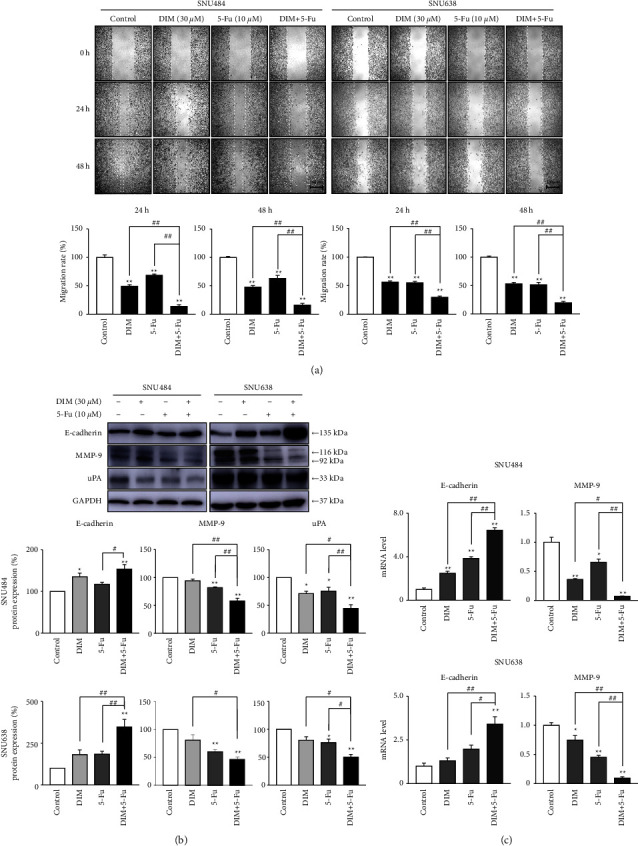
Combination treatment of DIM and 5-Fu diminished gastric cancer cell migration. (a) DIM and 5-Fu treatment inhibited cell metastasis. Cell metastasis was assayed using wound-healing. Cells were placed with 30 *μ*M DIM, 10 *μ*M 5-Fu, or a combination of the two drugs under 5% FBS. Migration rates were measured 24 h and 48 h (b) & (c) Combination treatment of DIM and 5-Fu regulated migration-related molecular E-cadherin, MMP-9, and uPA. Internal control was used as GAPDH. CONT, control; D or DIM, 3,3′-diindolylmethane; 5-Fu, 5-fluorouracil. Each point represents the mean ± standard error (SE). The gray value of bands was detected using ImageJ. ^*∗*^*p* < 0.05, ^*∗∗*^*p* < 0.01 compared to control; ^#^*p* < 0.05, ^##^*p* < 0.01 compared to DIM + 5-Fu.

Wound-healing assay was performed to test the inhibitory abilities of DIM and 5-Fu on the metastasis in GC cells. Cell migration rates were measured at 24 h and 48 h (Figure 3(a)). While the migration rate in SNU484 and SNU638 cells declined by approximately 50% 24 h and 48 h after treatment with DIM or 5-Fu alone, the migration rate reduced by approximately 70% 24 h and 48 h after DIM and 5-Fu treatment in GC cells. This result indicated that DIM and 5-Fu strongly decreases the migration rate of GC cells. Subsequently, we examined how the combined therapy of DIM and 5-Fu affects cell metastasis. Combined treatment of DIM and 5-Fu significantly promoted E-cadherin levels but suppressed MMP-9 and uPA levels in SNU484 and SNU 638 cells (Figure 3(b)). Moreover, combined treatment of DIM and 5-Fu considerably upregulated the E-cadherin mRNA and downregulated the MMP-9 mRNA (Figure 3(c)). Therefore, these findings suggested that combined treatment of DIM and 5-Fu significantly diminishes the migration ability of GC cells by regulating E-cadherin, MMP-9, and uPA.

### 3.4. DIM Enhances 5-Fu-Inhibited Akt Signaling in GC Cells

**Figure 4:**
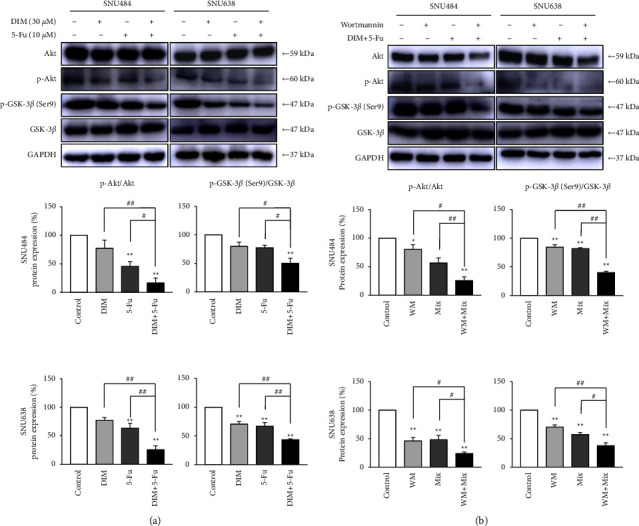
Combination treatment of DIM and 5-Fu inactivated the Akt signaling in gastric cancer cells. (a) The effect of DIM and 5-Fu treatment on the Akt signaling pathway. The Akt, p-Akt, p-GSK-3*β* (Ser9), and GSK-3*β* were measured after treatment with DIM (30 *μ*M), 5-Fu (10 *μ*M), or combined treatment for 48 h. (b) The effect of DIM and 5-Fu combination treatment with the presence of wortmannin on the Akt signaling pathway. Wortmannin (5 *μ*M) pretreatment for 2 h followed by treatment with DIM, 5-Fu, or combination treatment for 48 h Internal control was used as GAPDH. CONT, control; DIM, 3,3′-diindolylmethane; 5-Fu, 5-Fluorouracil; WM, wortmannin; mix, DIM + 5-Fu. Each point represents the mean ± standard error (SE). The gray value of bands was detected using ImageJ. ^*∗*^*p* < 0.05, ^*∗∗*^*p* < 0.01 compared to control; *p* < 0.05^#^, ^##^*p* < 0.01 compared to DIM + 5-Fu.

Akt kinase is a signal molecule for the typical PI3K effector in the PI3K/Akt signaling pathway, and its activation is associated with the pathogenesis of GC [35]. We evaluated the alterations in the Akt signaling and determined the suppressive GC cell growth's effect of DIM and 5-Fu. Combination treatment of DIM and 5-Fu considerably diminished the p-Akt, whereas no significant changes were seen in the Akt in SNU484 and SNU638 cells (Figure 4(a)). Furthermore, p-GSK-3*β* (Ser9), a downstream gene of Akt, greatly decreased after DIM and 5-Fu treatment. We further investigated whether the combined treatment of DIM and 5-Fu in the presence of wortmannin, a PI3K/Akt inhibitor, resulted in a more effective inhibitory effect in SNU484 and SNU638 cells. The combined treatment of DIM and 5-Fu significantly suppressed Akt, p-Akt, and p-GSK-3*β* (Ser9) levels in SNU484 and SNU638 cells with wortmannin treatment (Figure 4(b)), suggesting that wortmannin further accelerated the efficacy of the inhibitory effect of combination treatment of DIM and 5-Fu on the Akt pathway.

### 3.5. DIM Enhances 5-Fu-Inhibited Wnt Signaling in GC Cells

**Figure 5:**
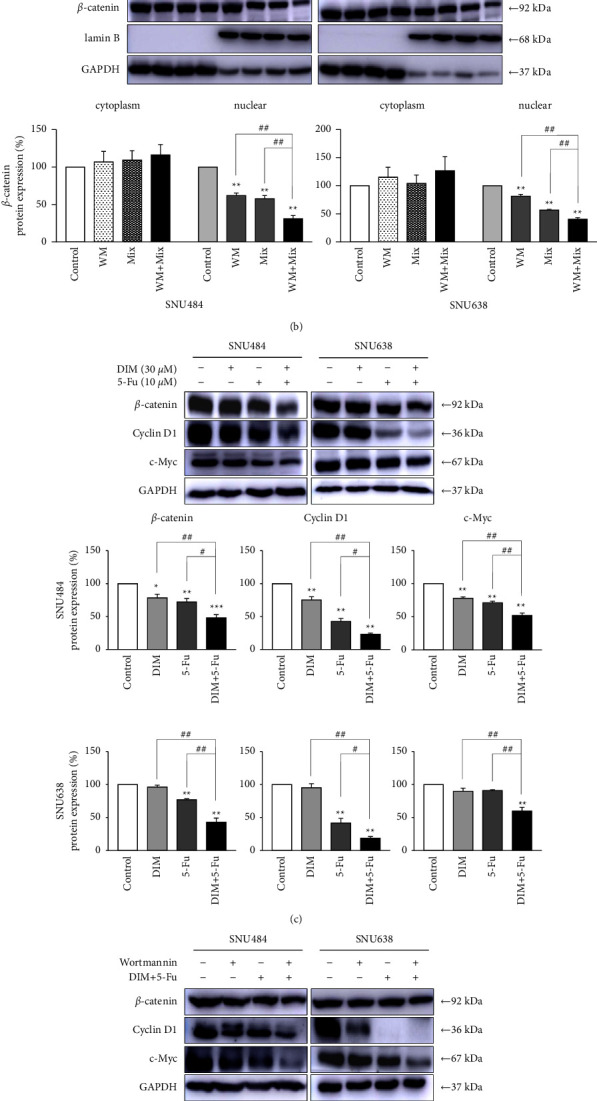
Combination treatment of DIM and 5-Fu inactivated the Wnt signaling in gastric cancer cells. (a) DIM and 5-Fu treatment decreased the nuclear *β*-catenin levels in GC cells. (b) DIM and 5-Fu treatment with or without wortmannin further decreased the nuclear *β*-catenin levels in GC cells. (c) & (d) Combination treatment of DIM and 5-Fu significantly inactivated the Wnt signaling. *β*-catenin, cyclin D1, and c-myc were detected after DIM (30 *μ*M), 5-Fu (10 *μ*M), or combination treatment for 48 h (d) combination treatment of DIM and 5-Fu with or without wortmannin significantly inactivated the Wnt signaling pathway. Lamin B and GAPDH was used as internal nuclear and cytoplasmic controls, respectively. CONT, control; D or DIM, 3,3′-diindolylmethane; 5-Fu, 5-Fluorouracil. Each point represents the mean ± standard error (SE). The gray value of bands was detected using ImageJ. ^*∗*^*p* < 0.05, ^*∗∗*^*p* < 0.01 compared with control; ^#^*p* < 0.05, ^##^*p* < 0.01 compared with DIM + 5-Fu.

As a downstream signaling pathway of PI3K/Akt signaling, the Wnt signaling pathway has been considered to play a critical role in cancer [38]. We further examined whether combination treatment of DIM and 5-Fu can regulate the Wnt singling pathway using western blotting. The main role of Wnt signaling stimulation is the transfer of *β*-catenin in the cytosol to the nucleus [42]. Hence, we separated the cytoplasm and nuclear protein to measure *β*-catenin nuclear translocation after combination treatment of DIM and 5-Fu. Despite no obvious change in the cytoplasm, the *β*-catenin levels in the nucleus significantly decreased after the combined treatment of DIM and 5-Fu compared to that after single treatment (Figure 5(a)). Furthermore, in the presence of wortmannin, combination treatment of DIM and 5-Fu dramatically inhibited *β*-catenin nuclear translocation (Figure 5(b)). Combined treatment with DIM and 5-Fu inhibited the *β*-catenin, cyclin D1, and c-Myc (Figure 5(c)). Moreover, combined treatment of DIM and 5-Fu significantly diminished the c-Myc and cyclin D1 in the presence of wortmannin (Figure 5(d)). These results suggested that combined treatment of DIM and 5-Fu not only inactivates the Akt signaling pathway through the Akt phosphorylation with p-GSK-3*β* but also inhibits the Wnt signaling through downregulating *β*-catenin in GC cells.

### 3.6. DIM Enhances 5-Fu-Inhibited Tumor Growth in Animal Models

**Figure 6:**
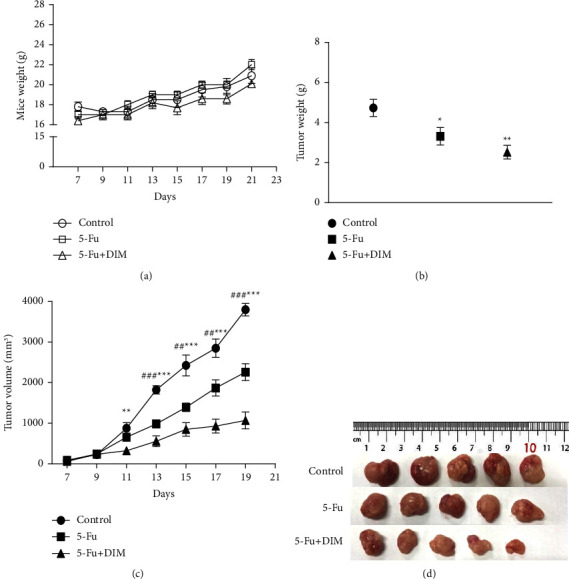
Combination treatment of DIM and 5-Fu diminished tumorigenesis in a xenograft model. (a) Mouse body weight. (b) Tumor weight. (c) Tumor volume curve. (d) Image of tumors. CONT, control; D or DIM, 3,3′-diindolylmethane; 5-Fu, 5-Fluorouracil. All therapies were intraperitoneally injected once every 2 days. All data were shown as the mean ± standard error (SE). ^*∗*^, the DIM + 5-Fu treatment group compared to the control group; ^#^, the 5-Fu treatment group compared to the control group. ^*∗*^*p* < 0.05, ^*∗∗*^*p* < 0.01, ^*∗∗∗*^*p* < 0.001; ^#^*p* < 0.05, ^##^*p* < 0.01, ^###^*p* < 0.001.

**Figure 7:**
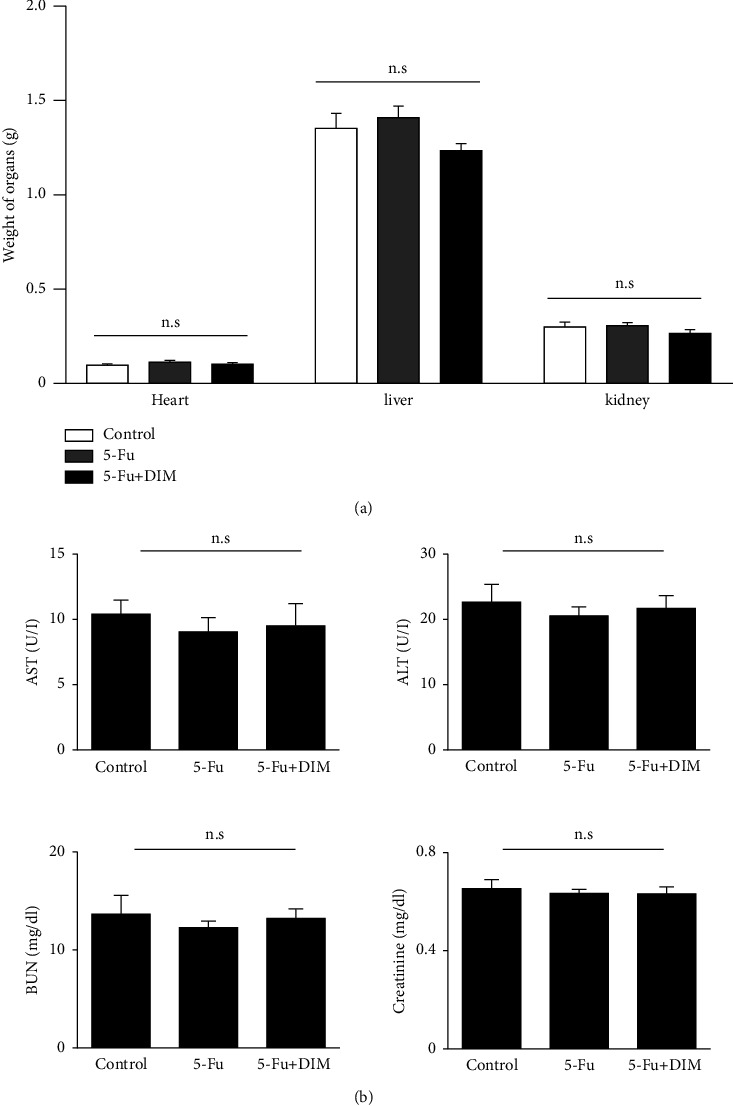
Toxic effects of DIM and 5-Fu treatment on a xenograft model. (a) Organ weight (i.e., heart, kidney, and liver). (b) Difference between the control and experimental groups in the serum levels aspartate transaminase and alanine aminotransferase (ALT), and nephrotoxicity indicators, BUN and creatinine. CONT, control; DIM, 3,3′-diindolylmethane; 5-Fu, 5-Fluorouracil. All data were presented as the mean ± standard error (SE).

To test the role of DIM and 5-Fu in tumorigenesis *in vivo*, xenograft mouse models were established. Referring to the previous study that revealed that DIM suppressed gastric tumorigenesis in a xenograft model [30], after subcutaneous injection with SNU484, the mice were classified into three groups, namely, the control, 5-Fu treatment, and DIM and 5-Fu combination treatment groups. After 2 weeks of treatment, tumor size was measured. In the DIM and 5-Fu combined group, the tumor weight, volume, and size were substantially inhibited compared to control or 5-Fu treatment group (Figures 6(b)–6(d)). To determine whether there was drug toxicity in mice, we evaluated the toxic effects of combination treatment of DIM and 5-Fu in xenograft mice *in vivo*. No significant differences in the body weight (Figure 6(a)), heart, liver, and kidney (Figure 7(a)) functions were observed between the groups. Serum biochemical analysis with liver function markers showed no significant differences among the three groups (Figure 7(b)). Regarding nephrotoxicity, BUN and creatinine (Cr) levels were examined and exhibited no differences among the groups. Overall, these results suggested that combined treatment of DIM and 5-Fu more effectively suppressed tumorigenesis without any toxic effects in the xenografted mouse model *in vivo*.

### 3.7. DIM Enhances 5-Fu-Inactivated Akt and Wnt Signaling Pathways In Vivo

**Figure 8:**
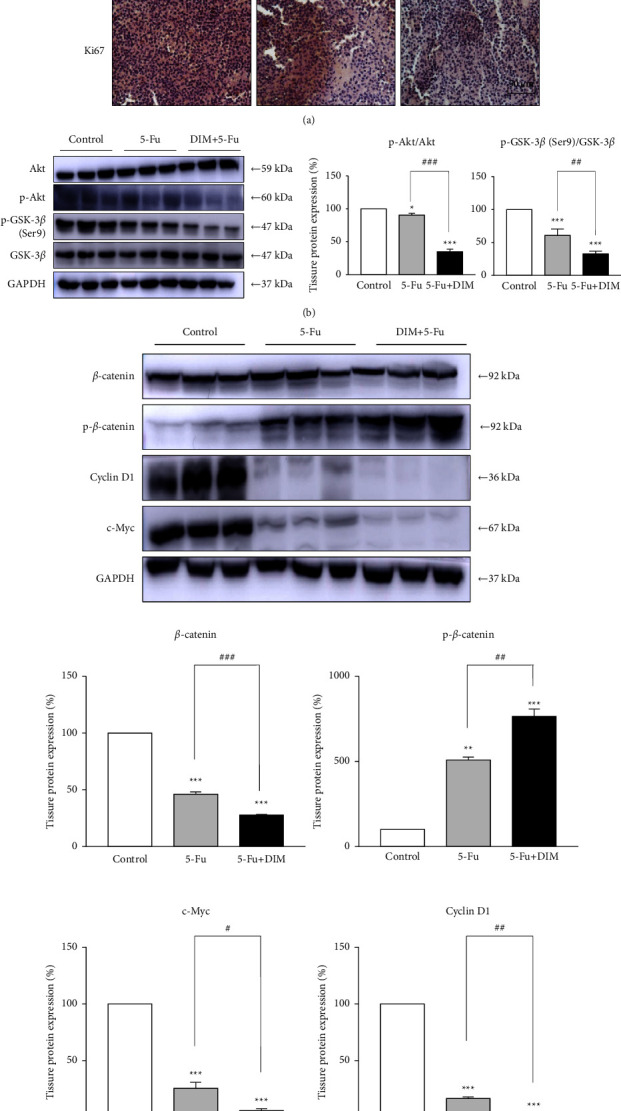
Combination treatment of DIM and 5-Fu inactivated the Akt and WNT/*β*-catenin signaling pathways *in vivo*. (a) Histological tumor sections and Ki67; arrow: necrosis (b) & (c) Akt, p-Akt, p-GSK-3*β* (Ser9), *β*-catenin, p-*β*-catenin, c-Myc, and cyclin D1 protein levels from mouse tissue were analyzed. Three samples were randomly selected in each group. Internal control was used as (b) DIM, 3, 3′-diindolylmethane;5-Fu, 5-Fluorouracil. The gray value of bands was detected using ImageJ. ^*∗*^*p* < 0.05, ^*∗∗*^*p* < 0.01, ^*∗∗∗*^*p* < 0.001 compared with control; ^#^*p* < 0.05, ^##^*p* < 0.01, ^###^*p* < 0.001 compared with DIM + 5-Fu.

Histological tumor sections revealed a remarkable amount of necrosis in the tumors after combination treatment of DIM and 5-Fu (arrow in Figure 8(a)). In the control group, it was seen that the tumor was growing vigorously, and it highly expressed Ki-67-positive cells. However, the 5-Fu and DIM and 5-Fu combined treatment groups showed a marked elevation in the quantity of apoptotic and necrotic cells and a decrease in Ki-67-positive cells. To further test whether combination treatment of DIM and 5-Fu inhibits the Akt and Wnt signaling pathways *in vivo*, we performed western blotting to measure the Akt and *β*-catenin in tumor tissues. The changes in Akt, GSK-3*β*, and *β*-catenin protein levels were similar to the *in vitro* experiment results. The p-Akt, p-GSK-3*β* (Ser9), c-Myc, and cyclinD1 levels were substantially diminished, whereas those of p-*β*-catenin were greatly increased in the DIM and 5-Fu combined treatment group *in vivo* (Figures 8(b) and 8(c)). These results indicated that DIM augments the anticancer effect of 5-Fu*in vivo* via the Akt/GSK-3*β* and *β*-catenin signaling pathways.

## 4. Discussion

GC is a common malignant neoplasm with a prominent mortality rate in South Korea [2]. Poor diagnosis and the limitations of therapy lead to high mortality of GC worldwide [3]. Therefore, GC remains a serious burden on global health due to the limitations of therapy, and the discovery of new chemotherapy strategies is urgently needed.

5-Fu has been diffusely used for more than 50 years as an antitumor drug in various tumors, especially in GC [43]. Presently, 5-Fu remains the clinically recommended treatment for GC [4, 44]. However, drug resistance, toxicity, and low-level response hampered the application of 5-Fu in clinical settings [13]. DIM was a natural compound extracted from cruciferous vegetables [45]. DIM has several beneficial biological activities against numerous cancers [20, 46]. In our previous experiments, DIM arrested the progression of GC in terms of cell apoptosis, migration, and metastasis [29, 30]. Additionally, recent studies revealed that DIM and 5-Fu have a synergistic effect on colon and cervical cancer [47, 48]. However, the antitumor effects of DIM with classic chemotherapeutics 5-Fu in GC are still indistinct. In this study, we selected two gastric cancer cell lines, SNU484 and SNU638. SNU484 and SNU638 have been established and reported as poorly differentiated adenocarcinomas [49]. Gastric adenocarcinoma is a malignant epithelial tumor that originates from the glandular epithelium of the gastric mucosa. Approximately 90% of gastric cancers are adenocarcinomas [50]. Poorly diagnosed gastric adenocarcinomas are susceptible to metastasis and can grow in lymph nodes even in the early stages [51]. According to NCCN treatment guidelines, systemic chemotherapy should be administered after partial surgical resection for patients with a high recurrence and metastasis risk [52]. Therefore, we have chosen SNU484 and SNU638 cells as poor adenocarcinoma markers to examine the therapeutic effects of an anticancer herbal component-based drug with chemotherapy for treating gastric cancers. We found that DIM greatly augments the anticancer effects of 5-Fu by suppressing proliferation and metastasis of GC cells. The combined treatment of DIM and 5-Fu more significantly inhibited cell viability than single-drug treatment. Similar results were observed in the cell colony formation growth. Moreover, we found that in cell cycle analysis, the sub-G1 phase, a maker of apoptosis, was significantly induced after DIM and 5-Fu cotreatment compared with that after single-drug treatment. Furthermore, annexin V–FITC staining data showed that the combination treatment induced apoptosis and necrosis compared to the single-drug treatment in GC cells. Members of the Bcl-2 family include Bax and Bcl-2. Furthermore, it is widely known that Bax's antiapoptotic function can compete with Bcl-2 [53]. Subsequently, essential proteins for apoptosis can be further activated by caspase-3 after Bcl-2 has been activated to trigger apoptosis [54]. These results are connected to the regulated expression of PARP and caspase-3, -7, -9. Combined treatment of DIM and 5-Fu improved the cleaved-forms and activated proteins that increase during apoptosis compared with single-drug treatment. The inhibitory effects of DIM on cell proliferation in breast [27] and colon cancers [25] were similar to those of our findings. Through this study, we found that the efficacy of 5-Fu further increased after DIM was added; combined treatment of DIM and 5-Fu significantly inhibited the cancer cell growth and induced apoptosis. This synergistic effect of 5-Fu and DIM was also shown in cancer metastasis and migration. In the wound-healing assay, combined treatment with DIM and 5-Fu notably inhibited cell migration after 24 h and 48 h, indicating that DIM substantially augmented inhibitory function of 5-Fu in GC migration. Moreover, combined treatment of DIM and 5-Fu significantly increased the levels of E-cadherin, which is referred to as the “suppressor of invasion” [55] and downregulated the protein levels of MMP-9 and uPA compared with DIM or 5-Fu single treatment. Moreover, combined treatment of DIM and 5-Fu suppressed the migration ability of GC cells through E-cadherin, MMP-9, and uPA, suggesting that DIM increases the anticancer activity of 5-Fu in GC cells. Therefore, these results offer substantial evidence that simultaneous treatment of DIM and 5-Fu synergistically suppresses GC cell growth and metastasis.

Akt, a PI3K downstream molecule, causes conformational changes through a series of actions, exposing the phosphorylation sites of Thr308 and Ser473 in the kinase domain and C-terminal domain, respectively [56], which promotes the cancer cell growth and provides resistance to cell apoptosis [34]. The phosphorylated Akt protein regulates a diversity of cell death pathways and cell-cycle transition [33]. Several studies have shown that chemotherapy reagents, such as 5-Fu and cisplatin, induce drug resistance by activating p-Akt and decreasing chemotherapy sensitivity in GC [57–59]. Less than 25% of patients with GC respond after 5-Fu treatment, and 5-Fu combined with other anticancer drugs only increased the response rate to 30%–50% [60, 61], suggesting that patients with advanced GC demonstrate resistance to 5-Fu-based chemotherapies. Therefore, the reduction of p-Akt expression appears to stimulate apoptosis and reduce the cell growth and chemotherapeutic resistance in GC [62, 63]. Furthermore, it has been reported that DIM inhibits Akt activity in many cancers, including GC [64]. In this study, we found that DIM and 5-Fu decreased the protein expression of p-Akt and p-GSK-3*β* (Ser9), and the combined treatment of the two further decreased the expression of p-Akt and p-GSK-3*β* (Ser9). In the presence of wortmannin, a nonspecific covalent PI3K inhibitor [65], combined treatment of DIM and 5-Fu markedly suppressed p-Akt and p-GSK-3*β* in GC cells. Therefore, these data suggested that DIM and 5-Fu inactivated Akt by dephosphorylation and may cause activation of GSK-3*β* via phosphorylation.

The Wnt signaling pathway, a downstream pathway of Akt, seems to be involved in breast carcinoma [66], lung cancer [67], colorectal cancer [68], and GC [69, 70]. *β*-catenin is working as a main mediator of the canonical Wnt pathway [71]. When the Wnt signaling pathway is activated, *β*-catenin accumulates and translocates into the nucleus to bind with T cell factor, acting as a transcriptional activator to trigger downstream target genes [42]. Numerous studies have shown that Akt activation caused the inactivation of GSK-3*β*, which repressed the degradation of *β*-catenin [72–75]. In this study, combined treatment with DIM and 5-Fu decreased *β*-catenin levels in the nuclei of GC cells. Our findings are consistent with those of previous studies, which reported that the activation of GSK-3*β* via the inactivation of Akt inhibits the translocation of *β*-catenin from the cytoplasm to the nucleus, suggesting that combination treatment of DIM and 5-Fu inhibits Akt activity, which activates GSK-3*β* and thereby induces the degradation of *β*-catenin in GC cells. Furthermore, the decreased amount of *β*-catenin caused by DIM and 5-Fu was further accelerated by adding wortmannin. The PI3K inhibitor, wortmannin, considerably prevented the phosphorylation of Akt and GSK-3*β*, which subsequently diminished the nuclear localization of *β*-catenin and therefore accelerated the tumor-inhibiting effect of DIM and 5-Fu. Cyclin D1 and c-Myc, as downstreams of Wnt, are the main participants of cell-cycle progression [76], in which aberrance has been associated with cell proliferation and apoptosis [77, 78]. DIM and 5-Fu treatment considerably diminished cyclin D1 and c-Myc compared to single treatment alone, and wortmannin further strengthened this decrease in GC cells. These similar findings were also observed in animal *in vivo* experiments. Combination treatment of DIM and 5-Fu significantly reduced tumor size/weight and volume compared with single treatment alone in xenograft animal models. Additionally, histological examination of tumor sections showed that a significant amount of necrosis in the tumors of mice treated with combined DIM and 5-Fu treatment significantly decreased the protein expression of p-Akt and p-GSK-3*β* (Ser9) in xenograft tumor tissue. Furthermore, combination treatment of DIM and 5-Fu induced *β*-catenin degradation and significantly reduced c-Myc and cyclin D1 levels compared with 5-Fu treatment alone in xenograft tumor tissues. Therefore, combination treatment of DIM and 5-Fu inhibited tumorigenesis more effectively without any toxic effects *in vivo* through the Akt and Wnt signing pathways. Our results suggested that combined treatment with DIM and 5-Fu not only inactivated the Akt signaling pathway through the dephosphorylation of Akt and its downstream protein p-GSK-3*β* but also inhibited the Wnt signaling through inactivation of *β*-catenin in GC.

In conclusion, our study revealed that DIM enhances the 5-Fu-inhibited cell growth and induced apoptosis by targeting the Akt/GSK-3*β* and Wnt/*β*-catenin signaling pathways; moreover, DIM suppressed cell migration by regulating E-cadherin, MMP-9, and uPA in GC. These anticancer effects were verified in cell lines and animal experiments, thereby indicating that DIM contributes to enhance the efficacy of 5-Fu, which is a widely used as anticancer drug. Therefore, our results provide convincing evidence that simultaneous treatment with DIM and 5-Fu synergistically suppresses the GC cell growth and tumorigenesis and could be a new potential therapy for treating GC.

## Data Availability

The data presented in this study are available within the article.
